# 43. BUILDING ON GENETICS AND PATHOPHYSIOLOGY OF SCHIZOPHRENIA TO GUIDE DISCOVERY OF NEW TREATMENTS

**DOI:** 10.1093/schbul/sby014.178

**Published:** 2018-04-01

**Authors:** P Jeffrey Conn

**Affiliations:** Vanderbilt University Medical Center

## Abstract

**Overall Abstract:** Current treatments for schizophrenia (SCZ) are partially effective in treating positive symptoms, but many patients are refractory to available medications and they are ineffective in the treatment of negative symptoms and cognitive impairment. There is a tremendous need to develop novel approaches to the treatment of SCZ that have broader efficacy and fewer adverse effects than existing dopamine D2 receptor and mixed D2/5-HT2A antagonists. To achieve fundamental breakthroughs that provide efficacy for refractory patients and across multiple symptom domains, it will be critical to rely on rigorous studies in SCZ patients that guide development of novel treatment approaches. Major recent advances in the genetics, imaging, and molecular pathophysiology of SCZ point to new potential treatment strategies, and may guide patient selection and outcome measures. Speakers in this symposium will summarize examples of translational studies that may offer novel therapeutic approaches that have the potential to fundamentally change the standard of care for SCZ patients. Jeff Conn (Vanderbilt Univ., USA) will summarize recent genetic studies that point to loss of function mutations in two novel G protein-coupled receptors in SCZ patients, and the discovery of novel positive allosteric modulators for these receptors that are providing a strong proof of concept for advancing selective agents to clinical evaluation in patients, including those that bear these specific mutations. Brian Dean (Univ. Melbourne, Australia) will then review clinical studies showing that a subgroup of SCZ patients show a marked loss of cortical muscarinic M1 receptors and translational studies suggesting that selective activators of M1 receptors could provide benefits in treating this disorder. In addition, he will summarize changes in molecular cytoarchitecture in the cortex of subjects with SCZ that provide important insights in considering M1 activators as a treatment strategy. Clare Beasley (Univ. British Columbia, Canada), will review mounting evidence from postmortem tissue that implicates immune dysregulation and the complement system in SCZ and the potential utility of complement inhibitors in the treatment of this disorder. Finally, Anissa Abi-Dargham (Stony Brook Univ., USA) will discuss recent advances in the use of molecular imaging for discovery of patients with alterations in specific targets that may guide clinical development. Specifically, imaging studies suggest that nigro-striatal dopaminergic projections may play a more important role relative to mesolimbic dopaminergic projections than previously appreciated, and suggest a blunting of dopamine release in extra-striatal areas. These observations can be linked to clinical treatment response or particular domains of pathology and fit nicely with new data suggesting that activation of targets outlined in the first presentation selectively inhibit dopaminergic signaling in nigrostriatal but not in other dopaminergic pathways. In summary, this panel will highlight new therapeutic leads derived from novel insights gathered through imaging, genetic and molecular studies of SCZ patients.

